# Telemedicine and Infectious Diseases Practice: A Leap Forward or a Step Back?

**DOI:** 10.1093/ofid/ofz196

**Published:** 2019-04-20

**Authors:** Rima C Abdel-Massih, John W Mellors

**Affiliations:** Division of Infectious Diseases, Department of Medicine, School of Medicine, University of Pittsburgh, Pennsylvania

## Abstract

Infectious Diseases (ID) specialists pride themselves on performing a thorough history and physical exam, and developing a comprehensive diagnosis and management plan. A timely question is whether this tradition is at risk from the coming wave of telemedicine in clinical practice? It would not be if ID specialists embrace the changes ahead and leverage new technologies to enhance the efficiency and reach of their clinical practices. In this report, we highlight the opportunities and challenges offered by telemedicine for ID practice (Table 1).

## Teaser

The critical shortage of infectious diseases specialists limits access to vital expertise for clinical care, antimicrobial stewardship and infection prevention, particularly in sparsely populated regions. Telemedicine services have emerged as a practical solution to expand access, but infectious diseases practitioners must embrace this major change in clinical practice. This invited perspective provides insights into the progress and challenges of extending the reach of infectious diseases expertise through telemedicine

## SEISMIC SHIFTS IN HEALTHCARE

The healthcare delivery landscape is changing rapidly because of advances in data storage and communication technologies, including electronic health records, robotics, videoconferencing, high-speed internet (4G), smartphones, personal digital assistants, and wearable monitoring devices. In addition to these breakthrough technologies, there are several factors promoting the growth of telemedicine services: the ongoing physician shortage, mainly affecting rural communities; the aging baby boomer population, requiring a greater medical workforce; the push for value-based care at lower costs with better health outcomes; and the need to increase patient satisfaction. Patients are becoming more accepting of telemedicine services and prefer more timely and convenient care (i.e., at home). The ID specialty has many distinguishing features that are amenable to telemedicine.

## TELEMEDICINE AND THE NEED FOR INFECTIOUS DISEASES EXPERTISE

The Infectious Diseases Society of America (IDSA) endorses the use of telehealth for the practice of ID [1]. Many different modalities can be used to provide Tele-ID care: live audio-video consultation; asynchronous consultations also known as “store and forward” consultation or “e-consults”; physician-to-physician telephonic consultations; mobile health applications; and distance-based training and monitoring of specialty care teams. However, there is little information available on the relative advantages of one telemedicine modality over another. Live video consultations and physician-to-physician telephonic consultations are most akin to traditional clinical practice but are less efficient than e-consults. Although there have been concerns that ID care via telemedicine is subpar to traditional in-person care, a recent single-center study showed that Tele-ID consultation was equivalent to in-person consultation [2]. In addition, providing access to ID care via telemedicine is likely to be beneficial compared to no care at all.

The need for ID expertise is increasing because of the shortage of trained physicians, especially in rural communities and smaller hospitals, where a traditional ID practice is difficult to sustain. Fueled in part by the opioid epidemics, pockets of human immunodeficiency virus (HIV) and sexually transmitted infection are emerging outside of metropolitan areas that require ID expertise. Patients with chronic infections, such as HIV or hepatitis C virus (HCV), organ transplant recipients, individuals on iatrogenic immunosuppression, and those harboring drug-resistant organisms living in underserved communities are required to travel or be transferred to tertiary centers or metropolitan hospitals to see an ID specialist, leading to prohibitive travel costs, inconvenience, high no-show rates, and the risk of delayed care and suboptimal outcomes. In addition, telehealth modalities may be the only viable solution for some hospitals and post-acute care settings to meet the new regulatory standards for antimicrobial stewardship implementation.

## TELE-INFECTIOUS DISEASES AT THE UNIVERSITY OF PITTSBURGH MEDICAL CENTER: A CASE STUDY

In response to a request for ID expertise at a rural hospital, the Division of Infectious Diseases at the University of Pittsburgh Medical Center (UPMC) established its first Tele-ID videoconferencing service in 2013 to provide both inpatient and outpatient care. Demand for this service grew rapidly across Western Pennsylvania over the next 5 years; today, Tele-ID services are provided to 13 different hospitals, both within and outside of the UPMC Health System. These 13 hospitals range in size from 30 to 317 beds, and 2 full-time ID physicians are sufficient to meet the demand for consultations. That only 2 ID physicians can cover 13 hospitals in a large geographic region provides a compelling rationale for Tele-ID services. In addition to the original live video consult service, e-consultations and physician-to-physician telephonic consultations are now available. This range of services has proven to be a very successful alternative to traditional in-person consultative care. The services are well received by local hospitals’ leadership, physicians, and, most importantly, patients. Recently, UPMC opened a new kidney transplant program at one of its larger regional hospitals. A Tele-transplant ID consultation service was put in place to evaluate and manage infections in organ transplant recipients.

**Table 1.** Tele-ID Opportunities and Majors Challenges

Opportunities	Challenges
In-patient consultations	Connectivity and EMR access
Live video consultation	Efficient workflow
Store-and-forward (e-consult)	Licensure and credentialing
Physician-to-physician telephonic consultation	Costs and reimbursement models
	Physician acceptance
Chronic Infection Management	
HIV	Implementation costs
HCV	Patient buy-in and outreach
TB	Physician acceptance
Antimicrobial Stewardship	Data access
	Reimbursement models
Outpatient Parenteral Antimicrobial Therapy	Continuity of care transition after discharge
	Coordination across care teams
	Costs and reimbursement models
Pre-exposure HIV Prophylaxis	Hard-to-reach populations
	Costs and reimbursement models

Abbreviations: EMR, electronic medical record; HCV, hepatitis C virus; HIV, human immunodeficiency virus; ID, infectious diseases; TB, tuberculosis.

## CURRENT AND FUTURE APPLICATIONS OF TELE-INFECTIOUS DISEASES

### Outpatient Settings

Studies have shown comparable outcomes for telemedicine and traditional practice for the management of chronic infections such as HIV and HCV infection [3]. Notably, in a US prison system study, patients with HIV infection managed by an ID specialist via telemedicine responded better to antiretroviral therapy compared with those managed by the prison primary care provider [4]. Telemedicine can also be used successfully to follow patients being treated for tuberculous infections and appears to be an acceptable alternative to directly observed therapy [3].

Outpatient teleservices can apply to other ID subspecialties such as pre-exposure prophylaxis (PrEP). A Tele-PrEP clinic was established at UPMC to reach and care for the lesbian, gay, bisexual, transgender, and questioning and Latinx youth in the Pittsburgh area. An HIV provider is now available online in a confidential setting during their dance lessons at a dedicated community center. Making care convenient for this at-risk population, without extra cost, should build a stronger connection with their care team and improve compliance with PrEP.

Another potential application is in Outpatient Parenteral Antimicrobial Therapy (OPAT) programs. Successful OPAT programs improve outcomes and decrease readmission rates [5]. Proper transition of care after hospital discharge, close monitoring for side effects and laboratory abnormalities, and response to therapy are important features of a Tele-OPAT program.

### Antimicrobial Stewardship

The Joint Commission now mandates that all hospitals and nursing care centers have antimicrobial stewardship programs. The Center for Medicaid and Medicare Services (CMS) also requires hospitals to comply with the Centers for Disease Control and Prevention’s antimicrobial stewardship core elements. Many rural hospitals, critical care access hospitals, and nursing homes lack the appropriate resources, workforce, and expertise to lead a successful antimicrobial stewardship program. The IDSA supports using telehealth to perform stewardship activities, provide education and supervision, and share tools and best practices [1]. Oversight by an ID physician or pharmacist via telehealth is also an acceptable alternative for the Joint Commission. Many healthcare systems are resorting to telehealth solutions to provide stewardship expertise across their facilities.

## CHALLENGES FOR  TELE-INFECTIOUS DISEASES SERVICES

A successful telehealth program needs to be economically sustainable and scalable. Choosing the right technology and leveraging existing information technology are critical to minimizing implementation expenses. Although more costly, devices including digital stethoscopes and high-definition cameras can improve the quality of a remote physical exam in a variety of care settings, and such technologies are likely to become more widely available at reasonable cost. Currently, the quality of video visits varies across locations and is critically dependent upon the quality of the local Internet connection and the expertise of the healthcare personnel facilitating the interaction. Operationally, variable workflows and the need for care coordination and scheduling are barriers to efficiency. For example, the need to login to different electronic medical record systems servicing several remote sites reduces physician efficiency.

Reimbursement is a major limiting factor for telemedicine implementation. Different revenue sources have been considered to support telehealth services such as grants, state funding, contract payments, and fee for service. The CMS has limited payments for telehealth services to beneficiaries in certain geographic areas located in authorized originating sites. However, in 2018, CMS recognized a broader range of “communication technology-based services” such as “virtual check-in,” professional evaluation of prerecorded images or video sent by pre-established patients, and telephonic or internet-based physician-to-physician consultation payment. This new position of CMS will broaden access to telemedicine services. In addition, up to 35 states and the District of Columbia have enacted telemedicine parity laws that require private payers to reimburse providers for telemedicine services similarly to in-person services. Medicaid payment varies in each state and can be determined on the State Medicaid agency website. Other hurdles for telemedicine include physician acceptance, medical licensure, credentialing, privileges, and liability. There are 24 states currently participating in the Interstate Medical Licensure Compact program, which will facilitate an expedited licensure process for physicians planning to practice in a different state.

## LOOKING AHEAD

Healthcare delivery is being redefined by advances in technologies, changes in policy and reimbursement, consumer demand for convenience, and the pervasive need to lower costs. The growth of virtual care is a disruption of the traditional patient-physician relationship, leaving no choice for hospitals and physicians but to embrace the change. Infectious Diseases specialists are no exception. The time is now for ID physicians to adopt telehealth technologies, develop new tools and more efficient workflows for patient care, and extend the reach of ID physicians to underserved populations. It is also time to implement cost-saving, value-based initiatives that the broad array of ID services and programs can offer to healthcare facilities. How soon and the extent to which telemedicine replaces traditional ID practice remains to be seen, but it is clear we are at turning point, and there is no stepping back.
